# Presence of Vaccine-Induced Antibodies Against *Leptospira* spp. Complicates the Diagnosis of Leptospirosis by the Microscopic Agglutination Test

**DOI:** 10.3390/vaccines13090956

**Published:** 2025-09-08

**Authors:** Katharina Gesa Schmitt, Michèle Bergmann, Hans van der Linden, Ahmed A. Ahmed, Reinhard K. Straubinger, Yury Zablotski, Katrin Hartmann

**Affiliations:** 1 LMU Small Animal Clinic, Centre for Clinical Veterinary Medicine, Faculty of Veterinary Medicine, LMU Munich, Veterinärstrasse 13, 80539 Munich, Germany; michele.bergmann@lmu.de (M.B.); y.zablotski@med.vetmed.uni-muenchen.de (Y.Z.); hartmann@lmu.de (K.H.); 2 Expertise Centre for Reference and Research on Leptospirosis, WOAH Reference Laboratory for Leptospirosis, UMC, Meibergdreef 39, 1105 AZ Amsterdam, The Netherlands; h.vanderlinden@amsterdamumc.nl (H.v.d.L.); a.ahmed@amsterdamumc.nl (A.A.A.); 3 Institute of Bacteriology and Mycology, Department of Veterinary Sciences, Faculty of Veterinary Medicine, LMU Munich, Sonnenstrasse 24, 85764 Oberschleissheim, Germany; r.straubinger@lmu.de

**Keywords:** leptospirosis, vaccination, 4-serovar vaccine, post-vaccination antibodies, MAT, *Leptospira* spp., immune response, immunization

## Abstract

Background: Leptospirosis is a potentially fatal infectious disease. Therefore, annual revaccination of dogs is recommended, but this can lead to diagnostic interference due to vaccine-induced antibodies. This study determined the prevalence of *Leptospira* spp.-specific antibodies in 97 healthy adult dogs revaccinated with a 4-serovar vaccine (Nobivac^®^ L4). Methods: Antibodies were measured with a microscopic agglutination test against 12 serovars before (week 0) and 2, 4, 12, 26, and 52 weeks after revaccination. Logistic regression analysis was performed to determine the presence of pre-revaccination antibodies. Mixed-effect logistic regression analyses and chi-squared tests were used to compare differences between antibodies against vaccine serovars and between vaccine and non-vaccine serovars at different time points. Results: Overall, 63/97 dogs (64.9%) had antibodies against vaccine serovars before revaccination. During the study period, antibodies against ≥1 vaccine serovars were detected in all 97 dogs (100.0%). The highest likelihood of detectable antibodies was present in weeks 2 and 4, but 71/97 dogs (73.2%) had antibodies persisting 52 weeks after revaccination. Of 97 dogs, 75 dogs (78.4%) even had antibodies against ≥1 non-vaccine serovars. Among those, 19/75 (25.0%) had a fourfold titre increase. Conclusions: These findings suggest that high levels of antibody titres against *Leptospira* spp. occur frequently and cross-reactivity against non-vaccine serovars is likely. The detection of vaccine-induced antibodies can therefore complicate the diagnosis of leptospirosis.

## 1. Introduction

Leptospirosis is a serious and potentially fatal bacterial infectious disease in dogs and other mammals, including humans [1]. *Leptospira* spp. is classified into more than 300 serovars [2], which are categorised in antigenically related serogroups based on their outer membrane lipopolysaccharide (LPS) antigens [3]. Transmission from dogs to humans is possible [4,5]. Thus, the protection of dogs is not only important for canine health but also for human health. The use of inactivated vaccines against leptospirosis has greatly reduced *Leptospira* spp. infections and urinary shedding in dogs [6,7,8,9,10]. In accordance with current guidelines, revaccination should be performed annually [11,12,13]. However, dogs with an incomplete vaccination history are still at risk of becoming infected [13]. In addition, infections are possible with a serovar not contained in a vaccine, specifically when using 2-serovar (L2) vaccines [14]. Thus, leptospirosis is still a common disease in many countries and a reliable diagnosis is essential for treating affected dogs and protecting owners and medical staff.

The reference method for diagnosing leptospirosis in dogs is the microscopic agglutination test (MAT), which detects antibodies against specific serovars of a defined *Leptospira* spp. panel. The limitations of MAT include the fact that antibodies might not be present in the early phase of infection and not all serovars are included in testing panels [4,13]. Even more problematic, antibodies induced by vaccination can be detected by MAT [15,16,17] and this can lead to misinterpreting results. Interference between vaccine-induced and disease-induced antibodies poses a significant problem when diagnosing leptospirosis. Studies have shown that antibodies are detectable 15 days [9] to 3 weeks [7] after the basic vaccination series. Studies in adult unvaccinated dogs demonstrated long-term presence of antibodies for 180 days after vaccination with different bivalent and multivalent vaccines [9,15]. Consequently, understanding how antibodies develop after vaccination is essential for the correct interpretation of MAT in vaccinated dogs in which leptospirosis is suspected. According to previous studies and current guidelines, a very high titre (≥800) against a serovar contained in the vaccine [18,19,20,21,22], antibodies against a non-vaccine serovar [23], or at least a fourfold increase in antibody titre in samples taken one or two weeks apart [12,13,17,18,19,20,21,22] have been used as criteria to detect infection.

The protective effect of vaccinations has been extensively studied [6,7,9,10,24,25]. Another study examined antibody development following primary immunisation [15]. But the variation in the detectability of different serovar antibodies over a year following regular vaccination in dogs is currently unknown. Furthermore, factors that may influence the detectability of antibodies after vaccination (such as previous vaccinations) have not yet been investigated. Therefore, the aims of this study were to evaluate (1) the prevalence of vaccine-induced antibodies using MAT in healthy adult dogs presented for annual revaccination, (2) the detectability and persistence of antibodies against vaccine and non-vaccine serovars after revaccination with an L4 vaccine over a period of 52 weeks, and (3) whether the number of previous regular vaccinations has an impact on antibody detectability.

## 2. Materials and Methods

### 2.1. Dog Population

This prospective study was approved by the government of Upper Bavaria, Germany (ethical approval code: ROB-55.2-2532.Vet_03-18-33). Only healthy adult dogs with bodyweights over 5 kg which had been vaccinated according to the current guidelines [13,26] and manufacturer’s instructions and had not received immunosuppressive drugs within 4 weeks prior to revaccination were included (*n* = 99). The vaccination requirements involved having previously received 2 vaccinations 3 to 4 weeks apart with a 4-serovar vaccine (mainly Nobivac^®^ L4, MSD Animal Health, Boxmeer, The Netherlands) and at least 1 revaccination with the same vaccine after one year (considered basic immunisation plus one revaccination, defined as “primary vaccination”). The majority of dogs (*n* = 70) had received annual revaccination at least once. The most recent vaccination was required to have been administered within 12 to 15 months before enrolment into this study. Dogs were considered healthy if they had no history of pre-existing disease, an unremarkable medical history, including normal behaviour, eating, drinking, defecation and urination, and an unremarkable physical examination. Dogs were excluded if they became ill (*n* = 1) or required immunosuppressive therapy (*n* = 1) at any time during the study period.

Dogs were presented on week 0 (day of yearly revaccination), as well as 2, 4, 12, 26, and 52 weeks (±2 days) after vaccination, to the same clinician who took their medical history, and performed physical examinations and serum sampling from the left or right saphenous vein at each time point. This resulted in 582 individual serum samples. Serum samples were stored at −80 °C until analysis via MAT. At week 0 and week 52, each dog received revaccination against canine leptospirosis with a 4-serovar vaccine (Nobivac^®^ L4, MSD Animal Health, Boxmeer, The Netherlands), which contained *Leptospira interrogans* serogroup Canicola serovar Portland-Vere, serogroup Icterohaemorrhagiae serovar Copenhageni, serogroup Australis serovar Bratislava, and *Leptospira kirschneri* serogroup Grippotyphosa serovar Dadas. The vaccine was administered subcutaneously on the left or right lateral abdomen. Vaccine-associated adverse events (VAAEs) were monitored via physical examinations (swelling, irritation or similar symptoms at the site of infection) and recorded by both the clinician and the dog owner at each time point during this study. A detailed medical history (specific questions regarding general behaviour or eating habits, etc.) was taken to complement the monitoring. In addition, owners were contacted three days and again two weeks after each vaccination to report any VAAEs.

### 2.2. Microscopic Agglutination Test

*Leptospira* spp. antibodies were measured using MAT. Testing was performed according to applicable standards [27]. Briefly, 50 µL of phosphate-buffered saline (PBS) and 10 µL of serum were added in a well of a 96-well flat-bottom plate. A dilution series of 10 to 640 was created over 6 wells and live *Leptospira* spp. organisms (25 µL) were added to each well. Live reference strains of 12 serovars belonging to 12 serogroups were used (Table 1). For the purposes of this study, the cut-off titre was ≥10 and referred to as “detectable antibodies” from this point onward. Negative and positive controls were included in each run.

Plates were incubated for 2 h at 30 °C and the results were then read out with a dark-field microscope by the same person. All samples were assigned individual numbers so that it was only possible to identify the time point in the course of this study at which the sample was drawn, but not the individual dog. The highest dilution level of serum in which at least 50% of leptospires still showed agglutination was assessed as the antibody titre for the respective sample and the corresponding serovar.

### 2.3. Statistical Analysis

Statistical analyses were performed using the statistical software R version 4.4.0. A total of 4 vaccine and 8 non-vaccine *Leptospira* spp. serovars were visualised as bootstrapped means and 95% confidence intervals. Mixed-effect logistic regression analyses were used to evaluate differences between different serovars at the same time point and between different time points within a particular serovar. Titres of vaccine serovars and non-vaccine serovars were compared with 2-sample chi-squared tests at every time point. A *p*-value < 0.05 was considered significant for all analyses.

Interactions between antibodies against vaccine serovars at certain time points and dogs’ age were examined using mixed-effect negative binomial regression models. In addition, mixed-effect logistic regression models were used to study factors associated with the presence of pre-revaccination antibodies, the persistence of antibodies until week 52 after revaccination, and the response of dogs to revaccination in terms of a fourfold increase in antibody titre against *Leptospira* spp. vaccine serovars. Investigated factors for the last three aspects included age, sex, weight, housing environment, a history of being abroad, living in a single- or multi-dog household, and vaccination history. Age was divided into three categories (young adult, 1 to <3 years of age; mature adult, 3 years to senior; senior dogs were defined as being in the last 25% of a dog’s life expectancy [28,29]), according to the American Animal Hospital Association [30]. Weight was subdivided into four categories (≥5 kg to <10 kg; ≥10 kg to <20 kg; ≥20 kg to <30 kg; ≥30 kg). For factor analysis, univariable analyses were performed first and then significant factors were included in a multivariate analysis afterwards. Backwards selection was applied until only relevant predictors remained. Vaccination history was grouped into 5 categories (only primary immunisation, 1–2 revaccinations, 3–5 revaccinations, 6–9 revaccinations, ≥10 revaccinations every 12 months after primary immunisation). The Wilcoxon Mann–Whitney U-test for non-normally distributed data was used to investigate whether previous leptospirosis vaccinations influenced antibody presence before or the response to revaccination with respect to vaccine serovars.

## 3. Results

### 3.1. Antibody Levels Before and After Revaccination

The final study population consisted of 97 dogs following 2 exclusions due to illness or immunosuppressive therapy. The dogs were between 2 and 14 years of age and had an average weight of 19.7 kg (range 5 to 50 kg). There were 57 female (58.8%) and 40 male (41.2%) dogs. Most dogs lived in an urban area (*n* = 74, 76.3%), as opposed to a rural area (*n* = 23, 23.7%). About half of the dogs (*n* = 52, 53.6%) lived with at least one other dog, and 63.9% (*n* = 62) of all dogs had been abroad.

**Figure 1:**
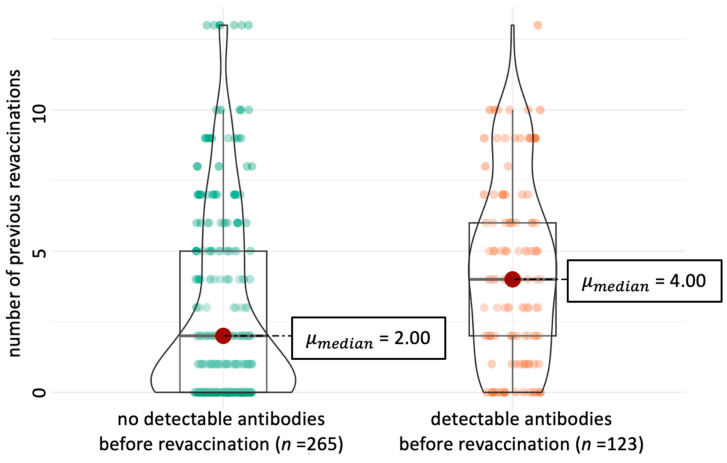
Dogs with detectable antibodies prior to revaccination (orange) had received significantly more previous vaccinations (*p* < 0.001, Mann–Whitney U-test). Dogs with no prior revaccination had only received primary vaccination before enrolment in this study.

Before revaccination (week 0), 63/97 dogs (64.9%) had measurable antibody levels against at least one vaccine serovar. Antibodies against at least one non-vaccine serovar were detected in 23/97 dogs (23.7%) (Table 2 and Table 3). Antibodies prior to revaccination (week 0) were significantly more often detectable (*p* < 0.001) with higher numbers of previous vaccinations (median = 4) than in dogs with fewer (median = 2) previous vaccinations (Figure 1, Table 4).

**Figure 2:**
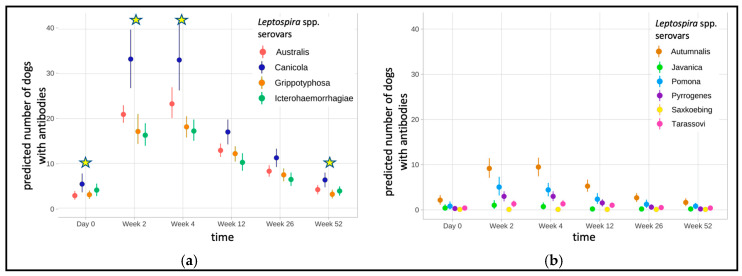
Predicted number of dogs with antibody titres against serovars tested at different time points (bootstrapped means and 95% confidence interval). More dogs had detectable titres against vaccine serovars after revaccination than against non-vaccine serovars: (**a**) Antibody titres are detected most commonly on weeks 2 and 4 against vaccine-serovars. Later, the number of dogs with antibodies decrease again, and are significantly lower in weeks 0 and 52 (time points at which the number of dogs differs significantly are marked with a star, originating from the mixed-effect model). (**b**) Titres against non-vaccine serovars showed no significant differences between time points.

The prevalence of dogs with antibodies against at least one vaccine serovar after revaccination is shown in Table 4. During the study period, antibody titres from 10 to 320 were detected. Overall, antibodies were significantly more often detectable in weeks 2 and 4 than at any other time point (*p* < 0.001). In contrast, antibodies were significantly less often detected in weeks 0 and 52 (*p* < 0.029) (Figure 2a).

Antibodies against at least one non-vaccine serovar after revaccination were detected ranging from 10 to 80 in 75/97 of the dogs (77.3%) (Table 5). There was no significant difference in the number of dogs with antibodies between time points (Figure 2b).

Comparisons between antibodies against vaccine and non-vaccine serovars were compared using mixed-effect logistic regression analysis and are reported in Table 6. Antibodies against vaccine serovars were significantly more likely to be detected in dogs than antibodies against non-vaccine serovars (*p* < 0.001).

**Figure 3:**
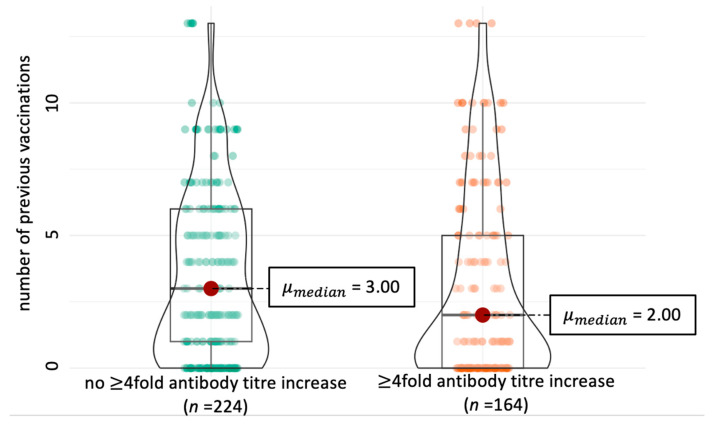
Association between vaccination history and fourfold antibody titre increase against revaccination. Significantly more dogs with fourfold antibody titre increases (*p* = 0.03, Mann–Whitney U-test) were detected in the group of dogs with fewer previous revaccination series (orange). No previous revaccinations indicate that the dogs were only given primary vaccination and no further revaccinations before being enrolled in this study.

In all dogs, an increase in antibody titres against at least one vaccine serovar was detectable and occurred mostly between week 0 and week 2 after revaccination (96/97 dogs, 99.0%). All dogs with detectable antibodies against non-vaccine serovars (75/97 dogs) also had detectable increases in antibody titres against non-vaccine serovars. The odds of at least a fourfold antibody titre increase were significantly higher in vaccine serovars than in non-vaccine serovars (*p* < 0.001). A fourfold increase was also significantly more often detectable from week 0 to week 2 compared to other time points (*p* < 0.001). In addition, dogs with 2 previous vaccinations additionally to their primary vaccination or less were significantly more likely to develop at least a fourfold antibody titre increase (*p* = 0.030) compared to dogs with more previous vaccinations (Figure 3). A detailed analysis can be found in Table 7.

Further analyses regarding factors associated with the presence of antibodies prior to revaccination and antibody response to revaccination can be found in the Supplementary Materials.

### 3.2. Vaccine-Associated Adverse Events

Overall, adverse events occurred only in 1/97 dogs (1.03%), and thus in 1/194 revaccinations (0.52%). Lethargy which lasted for 2 days was reported in a single dog after its second vaccination within this study. No injection-site reaction such as local swelling or pain was recorded.

## 4. Discussion

Dogs infected with *Leptospira* spp. pose a significant zoonotic risk. Therefore, the regular use of vaccines which do not only protect against disease, but also prevent shedding is essential. Since 2009, in Germany, *Leptospira* spp. vaccination has been considered by the Ständige Impfkommission Veterinärmedizin a core component of preventive health care in dogs [26] and, accordingly, many dogs are vaccinated on a regular basis. Previous studies investigated the protective effect of vaccination against *Leptospira* spp. and have mainly been performed on young Beagles in an experimental setting [6,7,9,10,24,25]. It is well known that the presence of antibodies does not necessarily indicate the vaccine’s efficacy, as even low titres or seronegative dogs were protected in experimental studies [6,10]. However, it is not only important to know whether and for how long vaccines provide protection, but also whether the vaccination interferes with diagnostics. This is the first study that investigated how *Leptospira* spp. antibodies can be detected in regularly vaccinated dogs over the course of one year using MAT. The present study focused exclusively on the detectability of antibodies for *Leptospira* spp. after L4 vaccination. This has clinical relevance as induced antibodies can complicate the interpretation of diagnostic tests for leptospirosis, i.e., MAT. Martin and colleagues previously investigated antibody responses in 32 dogs following vaccination [15] for 56 weeks, but did so in dogs over 15 kg bodyweight which had not been vaccinated for at least one year. Indeed, the majority of the dogs (*n* = 21) had never been vaccinated before. Therefore, the studied dogs all received basic immunisation during the study period. In contrast, the present study only included dogs (minimum bodyweight of 5 kg) which had received basic immunisation plus at least one revaccination (referred to as “primary vaccination” further on) with an L4 vaccine and have been vaccinated annually since then.

Interestingly, even before their yearly revaccination (week 0), 63/97 dogs (64.9%) had detectable antibodies against ≥1 vaccine serovars (at least 12 months and maximum 15 months after their last vaccination). In a previous vaccination field study by Martin and colleagues in 2014 [15] in which *Leptospira* spp. antibodies in 32 healthy client-owned adult dogs were assessed, most dogs (31/32) had no antibodies before vaccination; however, only 11/32 dogs had been vaccinated in the past (as defined as inclusion criteria in the present study). Another (but very old) field study compared two groups of dogs (seven dogs that had been vaccinated with an L2 vaccine twice 12 months apart; and eight dogs that had never been vaccinated before) regarding antibody persistence against serovars Icterohaemorrhagiae and Canicola after vaccination [31]. In the group of previously vaccinated dogs, antibodies against both serovars (low titres < 20) were still detectable 12 months after vaccination in some dogs (exact numbers were not mentioned by the authors). In the other group, in all eight dogs, antibodies were not detectable anymore after 13 weeks after first vaccination. Thus, a reason for the high number of dogs with pre-revaccination antibodies in the present study might be their history of several regular previous (re)vaccinations in the past. Before enrolment, 70/97 dogs (72.2%) in the present study had received up to 13 yearly revaccinations and, indeed, according to the present statistical analysis, dogs with at least four previous revaccinations were more likely to have measurable antibodies on week 0 in comparison to dogs with fewer previous revaccinations.

After revaccination, all 97/97 dogs (100%) in the present study had antibodies against ≥1 vaccine serovars with titres ranging from 10 to 320. The highest measurable titres were detected against serovar Canicola within the first 4 weeks after revaccination. Comparable antibody titres were seen in a field study by Andre-Fontaine (2013), which included 102 healthy dogs that had received their last leptospirosis vaccination <3 months, 3–6 months, 6–12 months, or >12 months before a blood sample was drawn to be tested against serovars Canicola and Icterohaemorrhagiae by MAT [32]. In contrast, Martin and colleagues (2014) were able to detect markedly higher titres in their 32 vaccinated dogs, with highest titres (up to ≥800) in week 4 after first vaccination [15]. However, these dogs were vaccinated more often with an L4 vaccine within a short period of time (in weeks 0, 3, and 52) in contrast to the dogs in the present study, which were only vaccinated once within the first 4 weeks. This different vaccination interval likely caused higher titres in the study by Martin and colleagues (2014), which is supported by the fact that the highest antibody titres were mainly measured on week 4 after the second vaccination [15]. These findings are in agreement with the results of one experimental study in which 18 specific-pathogen-free Beagle dogs received two vaccinations 4 weeks apart and showed a markedly increase in antibody titres after each vaccination [6]. In addition, methodological peculiarities in distinct laboratories could lead to different test results when comparing the results of Martin and colleagues (2014) [15] to those of the present study. This has been shown by Miller and colleagues (2011), who had serum samples from vaccinated dogs analysed by up to five different laboratories [17]. The laboratories provided divergent results, with vaccination titres ranging between 50 and 6400 for the same samples. Nevertheless, both Martin and colleagues (2014) [15] and the present study show that high antibody titres in MAT can also be induced by vaccination, not only by an infection with *Leptospira* spp. Furthermore, all dogs in the present study showed persisting antibodies for all serovars (Canicola > Icterohaemorrhagiae > Grippotyphosa > Australis) contained in the vaccine up to 52 weeks. High antibody titres and long-persistent, vaccine-induced antibodies can therefore result in interference with diagnosing leptospirosis in regularly vaccinated dogs even after one year. Distinction between vaccine-induced antibodies and those produced by infection is not possible by MAT, which is still the most widely used test for the diagnosis of leptospirosis. Thus, a single high MAT titre cannot be used for diagnosing leptospirosis [15,18,32].

The present study also demonstrated that cross-reactions in MAT with serovars not contained in the vaccine can occur after vaccination. In total, 23 of 97 dogs (23.7%) had detectable antibodies (titres ≤ 80) against non-vaccine serovars (Autumnalis, Pomona, Tarassovi, Javanica, Pyrogenes, Ballum) before revaccination and 75/97 dogs afterwards. Although no hunting of rodents was reported, some dogs regularly drank from puddles. Thus, exposure to *Leptospira* spp. resulting in subclinical infections cannot be completely excluded either before or during the study period. However, it must be taken into account that antibody titres against non-vaccine serovars also increased within the first weeks after vaccination (in 65/75 dogs) and can therefore more likely be considered a response to vaccination. Furthermore, a wider cross-protection induced by shared LPS antigens of serovars belonging to the same serogroup has been demonstrated in gerbils [33] and dogs [34,35] as a desired effect in vaccine development. Cross-reactivity between serovars belonging to different serogroups has also been reported in other field studies [36,37,38]. For example, Spiri and colleagues (2017) evaluated MAT results before and after L4 vaccination [38]. The enrolled dogs (*n* = 48) received two vaccinations 3–4 weeks apart. Half of the dogs had antibodies against serovar Canicola before vaccination, but all dogs were negative for the other 13 serovars tested. After vaccination, all dogs had detectable antibodies against ≥1 vaccine serovars and 8–60% also had detectable antibodies against non-vaccine serovars (Bratislava, Autumnalis, Copenhageni, Pomona, Pyrogenes). Barr and colleagues (2005) showed that vaccines against serovars Pomona (serogroup Pomona) and Grippotyphosa (serogroup Grippotyphosa) also induced antibodies against serovar Autumnalis (serogroup Autumnalis) [36]. This serovar was also most frequently found among the non-vaccine serovars in the present study. Martin and colleagues (2014) observed highly variable antibody titres following vaccination against all serovars tested (Bratislava, Canicola, Grippotyphosa, Hardjo, Icterohaemorrhagiae, Pomona) [15]. Structural similarities due to surface antigens can occur not only between serovars of the same serogroup, but also across serogroups, leading to cross-reactivity between serovars of different serogroups [39]. The findings of the present study therefore suggest that the detection of antibodies against a non-vaccine serovar cannot necessarily be considered conclusive evidence of an active *Leptospira* spp. infection unless the detected non-vaccine serovar titres exceed the vaccine serovar titres.

According to the current guidelines, a fourfold antibody titre increase or higher is regarded as confirmatory for leptospirosis in clinically ill dogs [13]. To validly assess the increase in titres, the measurements have to be performed in the same laboratory, as results vary greatly between laboratories [17]. The present study, however, showed that a fourfold increase in titre can also be triggered by revaccination, as observed in 89/97 dogs (91.8%). This could be detected especially from week 0 to week 2 after revaccination (89/89 dogs), but, remarkably, a second fourfold titre increase in at least one vaccine serovar could also be detected from week 2 to week 4 (4/89 dogs, 4.5%). Some dogs (19/97, 19.6%) even had an increase of at least fourfold in antibody titres against a non-vaccine serovar from week 0 to week 2 after revaccination. Thus, the recording of a precise vaccination history is therefore crucial for the subsequent interpretation of MAT results.

To date, there has been little research on factors associated with antibody development following vaccination against canine leptospirosis. Studies on rabies vaccination, which is also an inactivated vaccine, showed that young dogs after their first vaccination are at higher risk of developing a lower antibody response to rabies vaccination [40,41]. Contrarily, in the present study, dogs with ≤2 revaccinations showed lower antibody titres over the study period and, consequently, were significantly more likely to develop an increase of at least fourfold in titres. Since all dogs included in the present study had been regularly vaccinated before enrolment, it can be assumed that annual revaccinations have a potentially significant influence on antibody response to future vaccinations. In conclusion, an increase of at least fourfold in antibody titres measured by MAT against vaccine or non-vaccine serovars does not necessarily indicate an infection in recently vaccinated dogs and, thus, is also not suitable for diagnosis in recently vaccinated dogs.

In this study, only 1/97 dogs showed adverse effects, which manifested as mild, self-limiting lethargy for 2 days after revaccination. No other VAAEs occurred. Spiri and colleagues (2017), on the other hand, reported higher rates of different VAAEs (23% and 10% of dogs) within 5 days after the first and second Nobivac^®^ L4 vaccinations [38], respectively, but these symptoms were also mild and temporary. According to Moore and colleagues (2005), young adult, small-breed neutered dogs that received multiple vaccines at a time were at greatest risk of developing VAAEs within 3 days after any vaccination [42]. The dog in the present study was a 3-year-old spayed female that had already been vaccinated with Nobivac^®^ L4 before and was not receiving any other vaccines at that time point. Other dogs that received additional vaccinations at the same time also did not develop VAAEs. Overall, the occurrence of VAAEs after Nobivac^®^ L4 vaccination appeared to be very rare.

According to the latest consensus statement on leptospirosis, a single diagnostic MAT should not be used for the diagnosis of leptospirosis. Rather, a combination of tests such as PCR (which additionally identifies the infecting serovar), IgM ELISA, or point-of-care tests should be used alongside MAT for the accurate early diagnosis of leptospirosis [13,16,43]. The findings of the present study confirm the importance of these recommendations as there is significant diagnostic interference caused by vaccine-induced antibodies when interpreting MAT alone. MAT tests must always be interpreted within the context of the vaccination history of the dog, particularly if vaccination was recent. Furthermore, the detection of non-vaccine serovar antibodies by MAT should be interpreted with caution as these too will increase following vaccination.

### Limitations

Only a single type of vaccine was used in this study; therefore, the conclusions cannot automatically be drawn for all leptospirosis vaccines available. Due to the volume of blood required, only dogs weighing at least 5 kg were included in this study. As antibody responses were greater in smaller dogs, it can be assumed that dogs weighing less than 5 kg might develop an even higher antibody response. Another limitation of the present study was that only 12 serovars (including the vaccine serovars) belonging to 12 different serogroups were included in the MAT panel. Less-common serovars from different serogroups might have also shown reactivity that was missed. Antibody development due to subclinical infections after outdoor exposure to *Leptospira* spp. cannot be excluded with certainty in the enrolled dogs.

## 5. Conclusions

All dogs in this study developed antibodies against ≥1 vaccine serovars after revaccination, and these remained high in most dogs for up to 52 weeks after revaccination. Moreover, antibodies even against non-vaccine serovars were detected after revaccination. Fourfold increases in antibody titres after revaccination could also be detected against both vaccine and non-vaccine serovars. Therefore, diagnosing leptospirosis in a regularly vaccinated dog can be challenging, and the complete dog’s vaccination history, alongside the time since the onset of symptoms, must be considered when interpreting MAT results, especially in recently vaccinated dogs, but even up to one year after the last vaccination.

## Figures and Tables

**Table 1:** Panel of strains and associated serogroups used for microscopic agglutination test.

Serogroup	Serovar	Reference Strain
Australis ^1^	Australis	Ballico
Autumnalis	Autumnalis	Akiyami A
Ballum	Ballum	Mus 127
Bataviae	Bataviae	Swart
Canicola ^1^	Canicola	Hond Utrecht IV
Grippotyphosa ^1^	Grippotyphosa	Moskva V
Icterohaemorrhagiae ^1^	Icterohaemorrhagiae	RGA
Javanica	Javanica	Veldrat Batavia 46
Pomona	Pomona	Pomona
Pyrogenes	Pyrogenes	Salinem
Sejroe	Hardjo	Hardjoprajitno
Tarassovi	Tarassovi	Perepelitsin

^1^ Serogroups that are an active component of the 4-serovar vaccine Nobivac^®^ L4 (MSD Animal Health, Boxmeer, The Netherlands).

**Table 2:** Dogs with detectable antibodies against vaccine and non-vaccine *Leptospira* spp. serovars before revaccination (week 0).

Serovars	Prevalence		Detectable Antibodies Against *n* Serovars			
	*n* (%)	95% CI ^1^	1 Serovar	2 Serovars	3 Serovars	4 Serovars
Vaccine serovars	63/97	0.550–0.737	24/63	25/63	11/63	3/63
	(64.9%)		(38.1%)	(39.7%)	(17.5%)	(4.8%)
Non-vaccine serovars	23/97	0.163–0.331	18/23	4/23	1/23	-
	(23.7%)		(78.3%)	(17.4%)	(4.3%)	

^1^ CI, confidence interval.

**Table 3:** Detection of antibodies against vaccine and non-vaccine *Leptospira* spp. serovars prior to revaccination (week 0).

	Serovar	Prevalence		Antibody Titre Range
		*n* (%)	95% CI ^1^	
Vaccine serovars	Icterohaemorrhagiae	30/63 (47.6%)	0.358–0.597	10–40
	Grippotyphosa	28/63 (44.4%)	0.328–0.567	10–20
	Canicola	35/63 (55.5%)	0.433–0.672	10–80
	Australis	26/63 (41.3%)	0.300–0.536	10–20
Non-vaccine serovars	Autumnalis	15/23 (65.2%)	0.458–0.847	10–20
	Pomona	4/23 (17.4%)	0.020–0.330	10–40
	Tarassovi	4/23 (17.4%)	0.020–0.330	10
	Pyrogenes	3/23 (13.0%)	−0.007–0.268	10
	Javanica	1/23 (4.3%)	−0.040–0.127	40
	Ballum	1/23 (4.3%)	−0.040–0.127	10
	Bataviae	0/23 (0.0%)	-	n.d. ^2^
	Hardjo	0/23 (0.0%)	-	n.d.

^1^ CI, confidence interval; ^2^ n.d., not detected.

**Table 4:** Healthy adult dogs and the association of previous vaccinations with pre-revaccination antibodies against *Leptospira* spp. vaccine serovars in a univariate and multivariate analysis using mixed-effect logistic regression models.

Variable	Category	Prevalence	Dogs with Pre-Revaccination Antibodies ^1^	Univariate Analysis			Multivariate Analysis		
				*p*-Value	Odds Ratio	95% CI ^3^	*p*-Value	Odds Ratio	95% CI
Previous re-vaccinations ^2^	primary immunisation	27/97	11/27	Ref. value ^4^	-	-	Ref. value	-	-
	≥1 to <3 vaccinations	25/97	15/25	0.443	0.502	0.165–1.530	0.482	1.940	0.640–5.890
	≥3 to <6 vaccinations	21/97	19/21	**0.001**	0.144	0.046–0.449	**0.001**	5.840	1.880–18.100
	≥6 to <10 vaccinations	19/97	16/19	**0.003**	0.215	0.068–0.068	**0.002**	4.740	1.510–14.900
	≥10 vaccinations	5/97	4/5	0.327	0.298	0.052–1.723	0.802	2.060	0.350–12.300

^1^ Against ≥1 *Leptospira* spp. vaccine serovars. ^2^ Vaccination history includes prior immunisation with any L4 vaccine, regardless of included serovars. ^3^ CI, confidence interval; ^4^ Ref. value, reference value. Bolded values indicate statistically significant results.

**Table 5:** Number of dogs (prevalence) with antibodies against ≥1 vaccine or non-vaccine *Leptospira* spp. serovars after revaccination.

Serovars	Number of Dogs (Prevalence, %, CI ^1^)						
	Overall	Week 0	Week 2	Week 4	Week 12	Week 26	Week 52
Vaccine serovars	97/97 (100.0%)	63/97 (64.9%) (0.555–0.744)	97/97 (100.0%)	97/97 (100.0%)	97/97 (100.0%)	92/97 (94.8%) (0.904–0.992)	71/97 (73.2%) (0.644–0.820)
Non-vaccine serovars	75/97 (77.3%) (0.690–0.857)	23/75 (23.7%) (0.202–0.411)	3/75 (4.0%) (−0.004–0.084)	22/75 (29.3%) (0.190–0.396)	20/75 (26.7%) (0.166–0.367)	10/75 (13.3%) (0.056–0.210)	20/75 (26.7%) (0.166–0.367)

^1^ CI, confidence interval.

**Table 6:** Predicted probabilities of detectable antibodies against vaccine and non-vaccine serovars calculated by mixed-effect logistic regression analysis. Antibodies against vaccine serovars were significantly more likely detectable at any time point.

Time Point	Vaccine Serovars ^1^ (95% CI ^2^)	Non-Vaccine Serovars ^1^ (95% CI)	*p*-Value	Odds Ratio (95% CI)
Week 0	27.0% (0.213–0.336)	3.3% (0.021–0.050)	**0.001**	11.0 (6.96–17.2)
Week 2	96.7% (0.945–0.980)	17.9% (0.139–0.227)	**0.001**	132.7 (78.18–225.2)
Week 4	97.0% (0.950–0.982)	19.1% (0.150–0.241)	**0.001**	137.7 (79.64–238.1)
Week 12	86.5% (0.818–0.901)	10.6% (0.079–0.141)	**0.001**	53.7 (36.31–79.3)
Week 26	64.8% (0.576–0.715)	5.0% (0.035–0.072)	**0.001**	34.8 (23.25–52.2)
Week 52	35.0% (0.284–0.422)	3.2% (0.020–0.048)	**0.001**	16.5 (10.49–26.1)

^1^ predicted probability of antibodies; ^2^ CI, confidence interval. Bold values indicate statistically significant differences.

**Table 7:** Healthy adult dogs with a ≥fourfold antibody titre increase against *Leptospira* spp. vaccine serovars after revaccination in a univariate and multivariate analysis using mixed-effect logistic regression models and Wilcoxon Mann–Whitney U-test.

Variable	Category	Prevalence	Dogs with ≥4-Fold Antibody Titre Increase ^1^	Univariate Analysis			Multivariate Analysis		
				*p*-Value	Odds Ratio	95% CI ^3^	*p*-Value	Odds Ratio	95% CI
Previous revaccinations ^2^	primary immunisation	27/97	25/27	Ref. value ^4^	-	-	Ref. value	-	-
	≥1 to <3 vaccinations	25/97	19/25	0.289	1.889	0.777–4.590	0.439	0.580	0.240–1.390
	≥3 to <6 vaccinations	21/97	17/21	0.152	2.190	0.856–5.610	0.159	0.470	0.190–1.170
	≥6 to <10 vaccinations	19/97	14/19	0.051	2.664	0.996–7.130	0.058	0.390	0.150–1.020
	≥10 vaccinations	5/97	5/5	0.912	0.609	0.126–2.950	0.999	1.050	0.220–5.150

^1^ Against ≥1 *Leptospira* spp. vaccine serovars. ^2^ Vaccination history includes prior immunisation with any L4 vaccine, regardless of included serovars. ^3^ CI, confidence interval; ^4^ Ref. value, reference value. Bolded values indicate statistically significant results.

## Data Availability

The authors confirm that the datasets analysed during this study are available from the corresponding author upon reasonable request.

## Supplementary Materials

The following supporting information can be downloaded at: https://www.mdpi.com/article/10.3390/vaccines13090956/s1, Figure S1: Predicted counts, or predicted frequency of occurrence, of antibodies against vaccine serovars A-D in individual age groups over the course of this study (mixed-effect negative binomial regression model); Figure S2: Association between vaccination history and detectable antibodies at week 52 after revaccination; Table S1: Signalment and history of dogs and their association with pre-revaccination antibodies against *Leptospira* spp. vaccine serovars in a univariate and multivariate analysis using mixed-effect logistic regression models; Table S2: Characteristics of dogs and their association with a ≥fourfold antibody titre increase against *Leptospira* spp. vaccine serovars in a univariate and multivariate analysis using mixed-effect logistic regression models and Wilcoxon Mann–Whitney U-test; Table S3: Characteristics of dogs and their association with no antibody titre increase against *Leptospira* spp. vaccine serovars in mixed-effect logistic regression models.
