# Supplementary figures and images for: Presence of Vaccine-Induced Antibodies Against Leptospira spp. Complicates the Diagnosis of Leptospirosis by the Microscopic Agglutination Test
Source: Vaccines (Basel). 2025 Sep 8;13(9):956. doi: 10.3390/vaccines13090956 (PMC12474255; doi:10.3390/vaccines13090956)

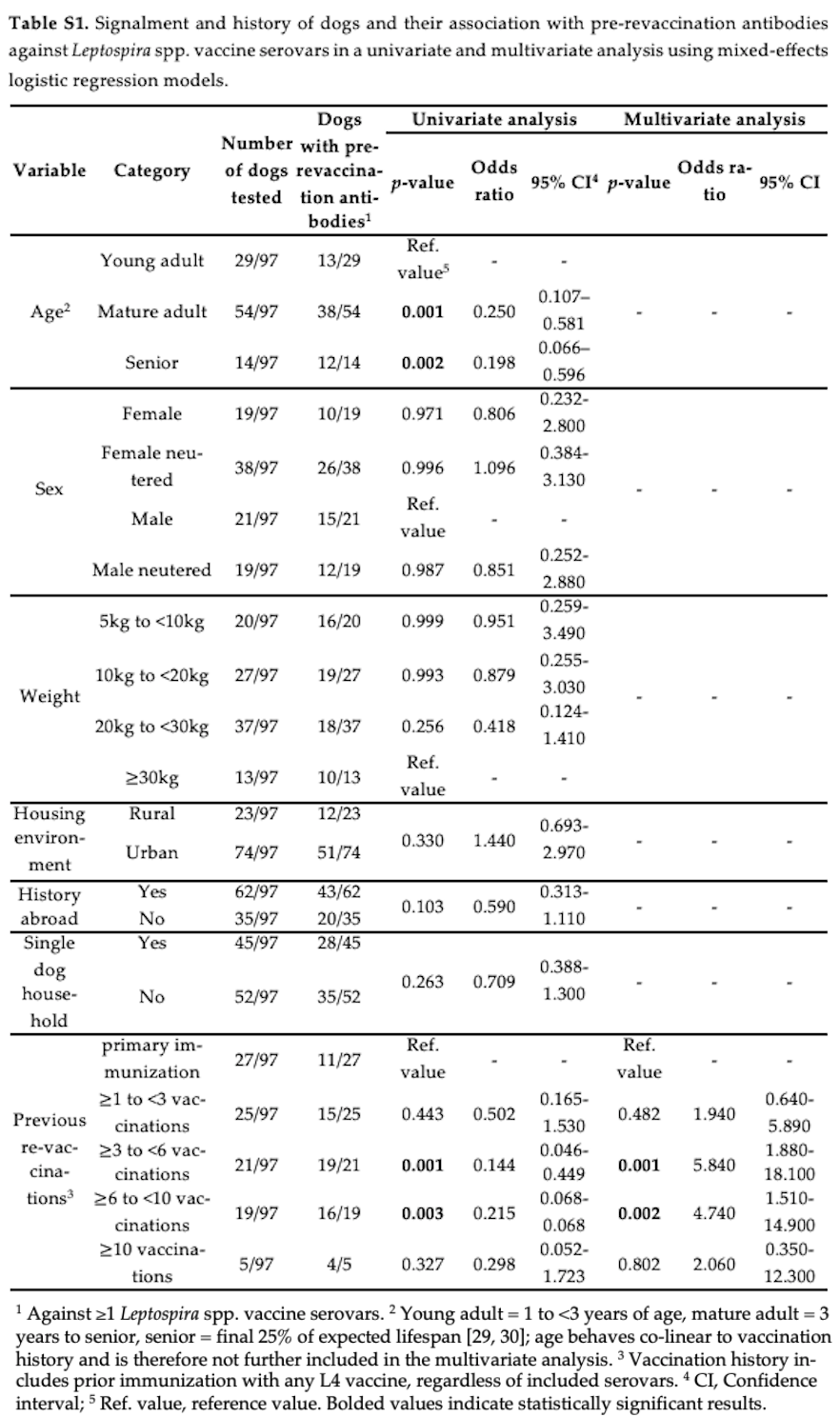

Supplement: Supplementary file 1 [file vaccines-13-00956-s001.zip › MAT Paper_supplementary Graphiken und Tabellen_proofread/Table S1.png]

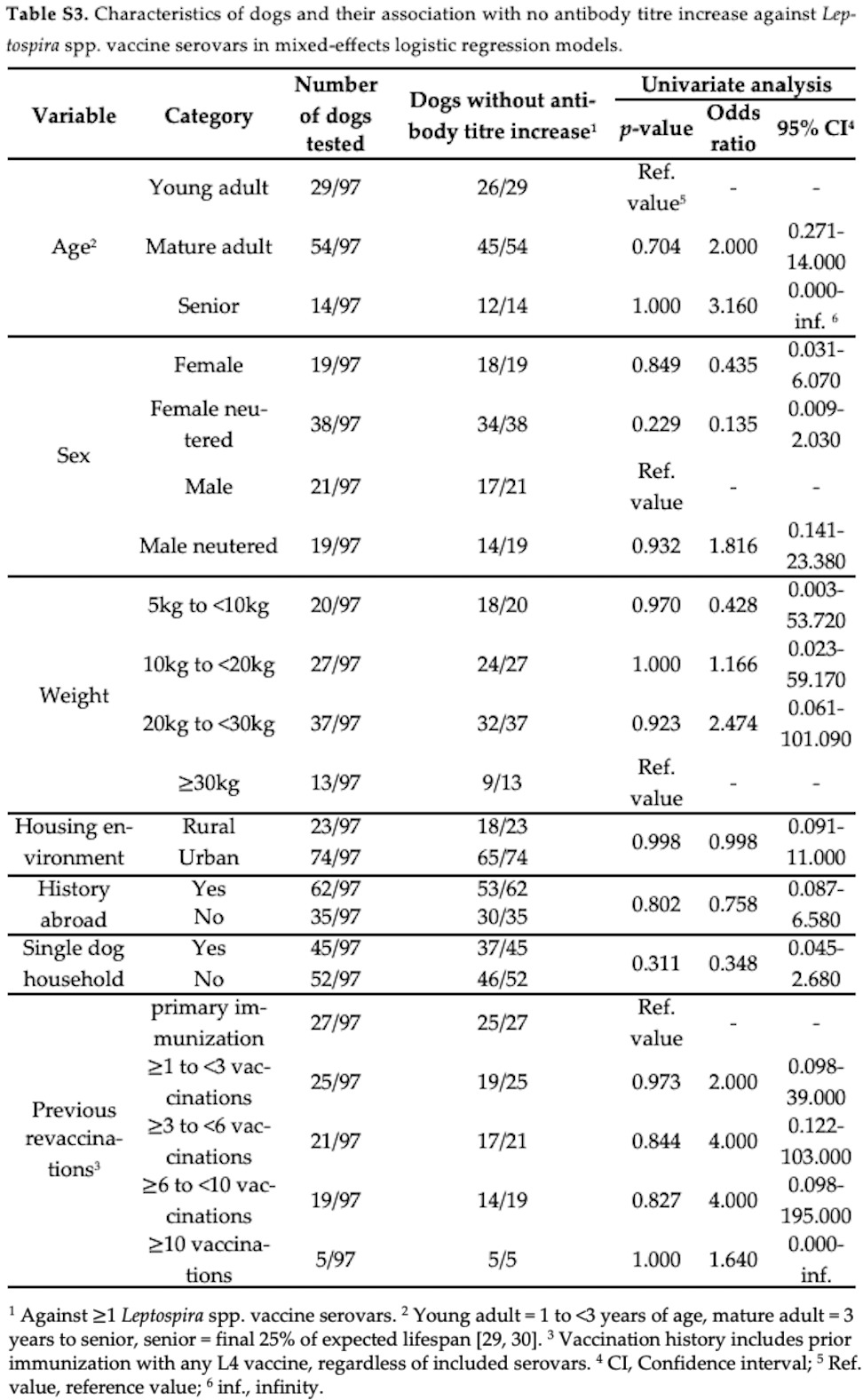

Supplement: Supplementary file 1 [file vaccines-13-00956-s001.zip › MAT Paper_supplementary Graphiken und Tabellen_proofread/Table S3.png]

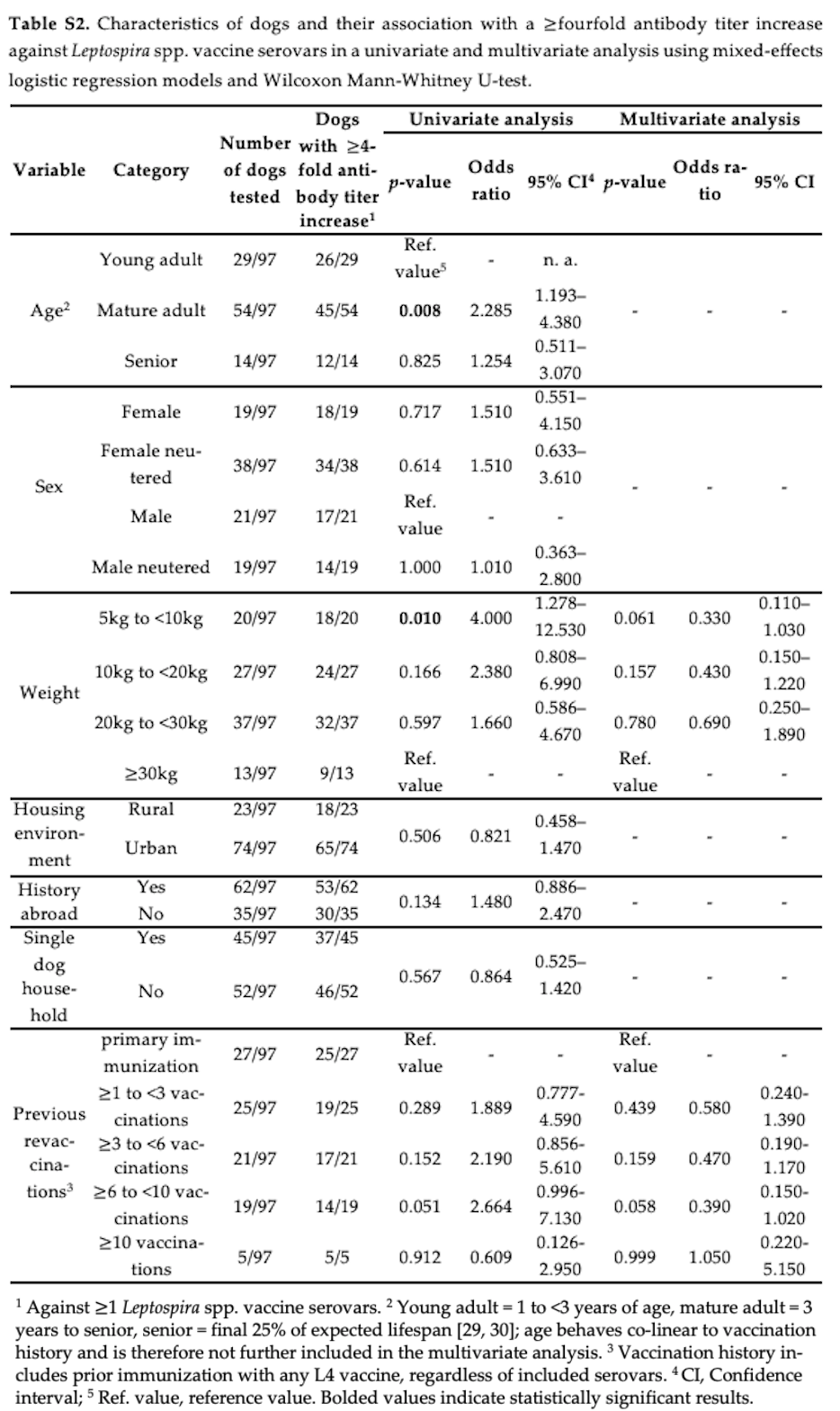

Supplement: Supplementary file 1 [file vaccines-13-00956-s001.zip › MAT Paper_supplementary Graphiken und Tabellen_proofread/Table S2.png]

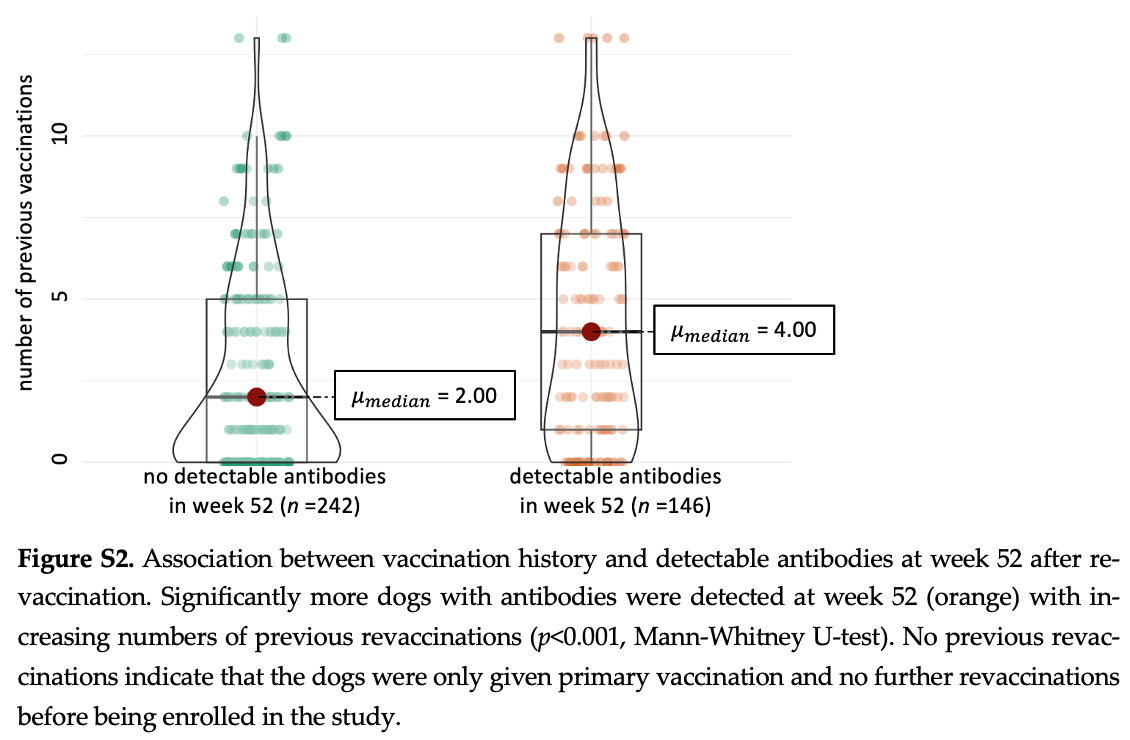

Supplement: Supplementary file 1 [file vaccines-13-00956-s001.zip › MAT Paper_supplementary Graphiken und Tabellen_proofread/Figure S2.png]

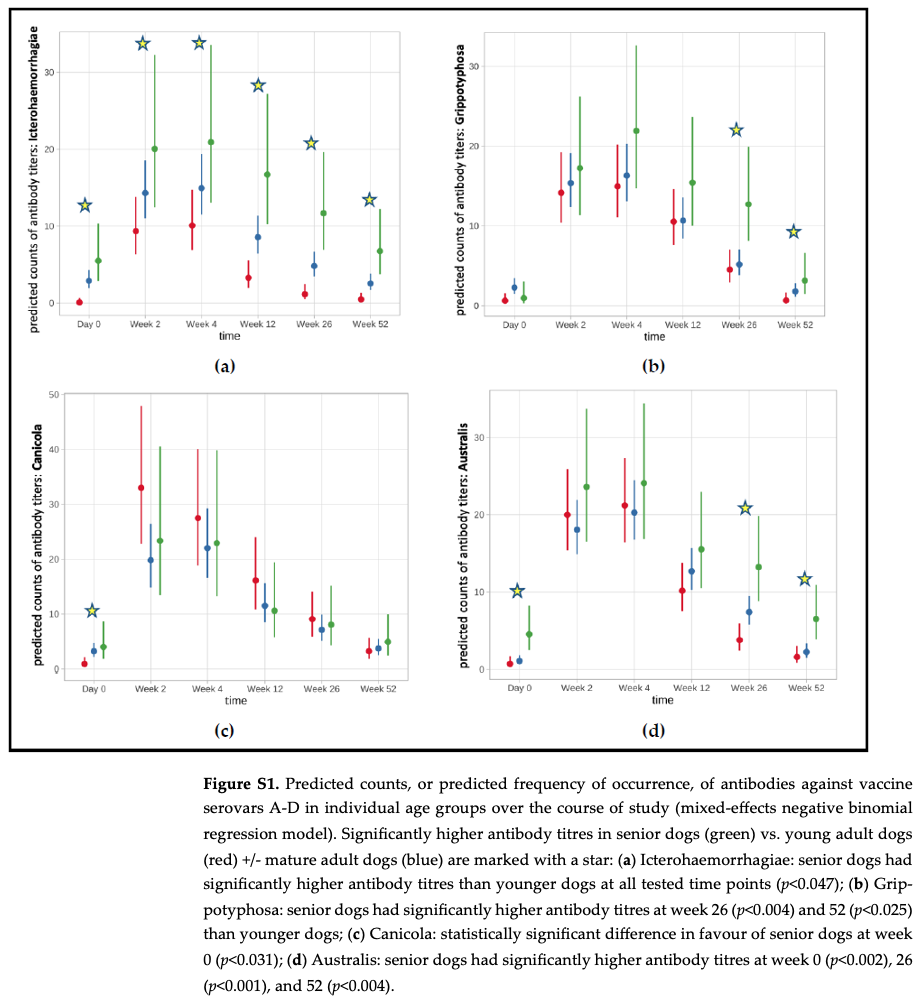

Supplement: Supplementary file 1 [file vaccines-13-00956-s001.zip › MAT Paper_supplementary Graphiken und Tabellen_proofread/Figure S1.png]
